# Supplementary material for: Effectiveness of Workplace Interventions to Improve Health and Well-Being of Health and Social Service Workers: A Narrative Review of Randomised Controlled Trials
Source: Healthcare (Basel). 2023 Jun 17;11(12):1792. doi: 10.3390/healthcare11121792 (PMC10298158; doi:10.3390/healthcare11121792)
Supplement: Supplementary file 1 [file healthcare-11-01792-s001.zip › healthcare-2405829-supplementary.pdf]

Table S1. Characteristics of the studies included in the review in chronological order.

1st Author and Year of Publication	Country	Study Population	Design	Follow-Up Time	Age at Baseline	Sex	Sample Size			Intervention				Control	Outcome	Results
							Interventio n	Control	Total	Type	Frequency	Duration	Place			
Barene 2014 [35] and 2022 [45]	Norway	Hospital employees	Cluster RCT	12 and 40 weeks	25 to 65 years. Mean age 46.1 ± 9.2 years	Women	37 football group and 35 Zumba group	35	107	Football matches or Zumba training	An hour session 2–3 times a week	40 weeks	Outside working hours using external training facilities	No intervention	Work ability, self-rated health, and emotional well-being<br><br>Work ability and self-rated health were measured by a single item<br><br>Emotional well-being was measured using 5 items of the RAND 36-item Health Survey	No significant changes in work ability were found in both intervention groups after 12 and 40 weeks.<br><br>Self-rated health was higher in Zuma group compared with control group at 40 weeks follow-up (mean difference 95%, *p* = 0.025) but no difference was found between football and control groups.<br><br>Emotional well-being at follow-up did not differ between intervention and control groups.
Booker 2022 [137]	Australia	Healthcare shift workers	Cluster RCT	6 months post-intervention	Mean age 34.7 ± 12.0 years	96% women	79	70	149	An educational and individualized program to manage sleep and the consequences of shift work	A one-on-one coaching session every two weeks<br><br>Bright light exposure for 20 min at a time during writing notes, breaks, or before driving home, frequent caffeine consumption (every 2 h until 6 h before sleeping), and a prescription of melatonin for those whose insomnia,	8 weeks	During work hours	8-week education on the benefits of a low glycemic diet	Sickness absence<br><br>Register data on sickness absence for 6 months	Sickness absence did not differ between intervention and control wards.

											sleepiness or sleep duration did not improve					
Brazier 2022 [46]	UK	Trainee anaesthetists	Individually RCT	10 months	Not reported	52.3% were women	74	79	153	22 text messages about factors associated with burnout	One text message every two weeks	10 months	Not applicable	No intervention	Well-being, burnout, and sickness absence<br><br>Well-being was assessed using 7-item work-related subscale of the Short Warwick-Edinburgh Mental Well-being Scale<br><br>Burnout was measured using 7-item Copenhagen Burnout Inventory<br><br>Number of self-reported sickness absence days in the past six months	Well-being, burnout, and number of sickness absence days did not differ between intervention and control groups.
Dahlgren 2022 [92]	Sweden	Registered nurses with <12 months' work experience	Individually RCT	Post-intervention and 6-month follow-up	Mean age 27.5 ± 5.3 years for intervention group and 27.0 ± 5.1 years for control group	84.7% of intervention group and 90.7% of control group were women	99	108	207	A recovery program based on cognitive–behavioural therapy and motivation consisting of proactive strategies for sleep and recovery regarding work stress and shift work	Three 2.5-h group sessions (2 week intervals)	4 weeks	During work hours	No intervention	Burnout<br><br>Burnout was measured with the 22-item Shirom-Melamed Burnout Questionnaire	The level of burnout was lower in intervention group than control group at post-intervention, but it did not differ between intervention and control groups at follow-up
Degen 2022 [115]	Germany	General practitioners and practice personnel	Cluster RCT	9 months after the intervention	Mean age 45.5 ± 12.3 years	85.4 % were women	129	139	268	Leadership skills training	Two 3.5 h leadership workshops at 2 week intervals, toolbox with materials and 9-month implementation phase supported by facilitators	9 months	Workplace	No intervention	Job satisfaction was measured with the Copenhagen Psychosocial Questionnaire	The intervention had no effect on job satisfaction

Errazuriz 2022 [113]	Chile	Healthcare workers	Individually RCT	Post-intervention (2 weeks after completing intervention) and 4 months post-intervention	Mean age 40.2 ± 11.7 years	97.1% of intervention groups and 100% of control group were women	N = 35 mindfulness-based stress reduction and N = 34 stress management course	36	105	Mindfulness-based stress reduction intervention.<br><br>Stress management course	A 2-h weekly session for mindfulness-based stress reduction.<br><br>Stress management course offered lectures about efficient coping strategies	8 weeks	On weekdays	No intervention	Job satisfaction<br><br>It was assessed using the Job Satisfaction Scale	Mindfulness-based stress reduction intervention statistically significantly improved job satisfaction at post-intervention but not at 4-month follow-up<br><br>Stress management course had no effect on job satisfaction
Fainstad 2022 [83]	USA	Resident physicians	Individually RCT	6 months	Mean age 29.4 ± 2.3 years	Women	50	51	101	Web-based professional group-coaching	Two 1-h group-coaching per week on a video-conferencing platform, unlimited anonymous written coaching, and weekly self-study modules	6 months (45 coaching sessions)	Weekdays at 7–8 PM	No intervention	Burnout (emotional exhaustion, depersonalization, and personal accomplishment) measured with the Maslach Burnout Inventory	Emotional exhaustion decreased in the intervention group by 3.26 ± 1.25 points while it increased by 1.07 ± 1.12 points in control group (*p* = 0.01 for between-group difference) during follow-up<br><br>Depersonalization and personal accomplishment did not differ between intervention and control groups
Fong 2022 [44]	Hong Kong	Nurses working at a university hospital	Individually RCT	10 days	21 years or older. 31.6% of intervention group and 34.2% of control group were older than 40 years	93.5% were women	39	38	77	Mindful colouring. Watching a 3-min instructional video and colouring mandalas for at least five working days or 100 min in 10 days	Daily	10 days	Workplace	No intervention	Mental well-being was assessed using a 7-item scale and burnout was measured using Maslach Burnout Inventory	The intervention had no effects on mental well-being and burnout (emotional exhaustion, depersonalization, and personal achievement)

Gerodimos 2022 [41]	Greece	Health professionals working at a hospital	Individually RCT	6 weeks	40–55 years. Mean age 44.4 ± 6.2 for intervention group and 44.3 ± 5.7 for control group	75% were women	25	25	50	Supervised workplace exercises consisting of stretching, strengthening, aerobic dance and balance exercises	Two 15–20 min sessions per day (2–3-h rest between them) for 5 days a week	6 weeks	Workplace	No intervention	Perceived well-being was assessed with the 5-item Subjective Vitality Scale	Perceived well-being improved in intervention group (*p* < 0.001) but no change was found in control group at follow-up. The difference between two groups was significant (*p* < 0.001)
Goktas 2022 [110]	Turkey	Day shift emergency nurses	Individually RCT	21 days	Mean age 29.8 ± 7.5 years for intervention group and 28.7 ± 6.9 years for control group	48.3% were women	30	30	60	Short motivational mobile phone messages	3 times per day	21 days	During work time	No intervention	Job satisfaction and compassion fatigue<br><br>A 5-item scale was used to measure job satisfaction and a 13-item scale to measure compassion fatigue	The intervention statistically significantly improved job satisfaction and reduced compassion fatigue<br><br>There were also significant differences in trauma and occupational burnout subscales of compassion fatigue between intervention and control groups
Ha 2022 [53]	South Korea	Nurses working rotating shifts	Cluster RCT	12 weeks	19–60 years. Mean age 27.6 ± 3.1 years for intervention group and 27.0 ± 3.7 years for control group	Women	30	27	57	A mobile wellness program to promote physical activity and improve sleep quality by providing Fitbit, online exercises via Zoom, online health coaching, and motivational text messages	Daily walking, online exercises for 1-h twice a week, and weekly online health coaching	12 weeks	Outside of work hours	Controls received a Fitbit for monitoring their activity and sleep	Wellness and fatigue<br><br>Wellness was measured using the 18-item wellness index<br><br>Fatigue was measured using the 19-item Multidimensional Fatigue Scale	The intervention statistically significantly improved wellness but had no effect on self-rated fatigue
Hata 2022 [96]	USA	Clinical physicians, nurse	Individually RCT	3 months	Not reported	96% were women	14	11	25	Self-facilitated discussion over	One meeting per month	3 months	2 meetings at hospital and one at	Self-facilitated discussion over	Burnout and work engagement	Overall burnout, emotional exhaustion and

		practitioners, and certified nurse midwives								dinner with a one-page discussion guide			a local restaurant	dinner on any subject	Emotional exhaustion and depersonalization were measured using a single item from the Maslach Burnout Inventory<br>Work engagement was assessed using the 9-item Utrecht Work Engagement Scale	depersonalization decreased, and work engagement increased at 3-month follow-up in both groups<br>There were no differences between intervention and control groups
Joshi 2022 [80]	USA	Healthcare professionals with distress (score 6 or higher based on 10-point Single Units of Distress Scale)	Individually RCT	1 and 3 months	Mean age 40 ± 11 years	82.5%were women	41	39	80	Transcendental meditation	The intervention group received 5 instruction sessions in 5 days and 20 min meditation twice a day	3 months	Outside working hours	Usual treatment (access to wellness resources)	Burnout (emotional exhaustion, depersonalization, and personal accomplishment) measured with the Maslach Burnout Inventory	The intervention reduced emotional exhaustion (difference between groups −5.4, p = 0.006)<br>Depersonalization was non-significantly lower (difference between groups –1.7, p = 0.08) and personal accomplishment was non-significantly higher (difference between groups 1.9, p = 0.11) in the intervention group
Lin 2022 [34]	China	Young dental professionals (dentists, dental nurses, and dental assistants)	Cluster RCT	3, 6, 9 and 12 months	Younger than 35 years. mean age 27.1 ± 5.3 for intervention group and 28.3 ± 6.0 for control group	66.8% were women	88	99		Participatory ergonomic changes consisted of adjustment in posture, reduction in repetitive movements, teaching proper tool usage, and work break arrangement and change in work time	A 45-min ergonomic lecture, and then an ergonomist spent 0.5 to 2 h 3 times a week util no changes were needed. A total of 235 ergonomic changes were made within a year	12 months	Workplace	Controls received a 45-min ergonomic lecture	Work ability<br>It was measured by 7-item Work Ability Index	Work ability index did not differ between intervention and control groups at 3- and 6-month follow-up<br>It slightly improved in intervention group compared with control group at 9

																months (between-group difference 0.53, 95% CI −0.02, 1.56, p = 0.055) and 12 months (between group difference 0.35, 95% CI −0.05, 1.31, p = 0.089) follow-ups
Pérez 2022 [61]	Spain	Nurses working at elder-care centres	Cluster RCT	Six weeks and 3 months after the intervention	25–56 years. Mean age 37 ± 9.1 years	89.6% were women	39	35	74	Mindfulness-based stress reduction training	An hour online session (total six sessions) plus two assignments (total 8) per week and one guided meditation audio every two weeks (total 5 audios)	Six weeks	Training was on a platform	No intervention	Burnout, compassion fatigue and compassion satisfaction measured using the 30-item Professional Quality of Life Scale	The intervention statistically significantly reduced compassion fatigue and burnout at 6 weeks and 3 months The intervention had no effect on compassion satisfaction
Sis Çeli 2022 [76]	Turkey	Nurses working in the COVID-19 clinics	Individually RCT	4 weeks	69.3% aged between 18 and 29 years and 30.7% aged 30 years or older	81.2% were women	51	50	101	Laughter yoga consisting of stretching, relaxation techniques, deep breathing exercises, and laughter exercises	A 40-min session via Zoom twice a week	4 weeks	Not reported	No intervention	Burnout It was measured using the 22-item Maslach Burnout Inventory	Overall burnout, emotional exhaustion, and depersonalization decreased, and personal accomplishment increased in intervention group than control group (p < 0.001, all)
Spilg 2022 [128]	Canada	Faculty physicians of a hospital's department of medicine	Individually RCT	3 and 6 months	Mean age 45.9 ± 9.1 for intervention group and 45.9 ± 10.1 for control group	37% were women	19	17	36	Stress management and resilience training	One 2-h mandatory in-person workshop and 24-week optional online program (one 45-min module per week for 4 weeks and one 10- to 15-min module per week for 20 weeks)	24 weeks	Workplace	No intervention	Subjective happiness was measured using the 4-item Subjective Happiness Scale	There was no difference in happiness between intervention and control groups at 3- and 6-months follow-ups
Taylor 2022 [42]	UK	Healthcare workers	Individually RCT	1.5 and 4.5 months after randomization	Mean age 40.4 ± 10.9 for intervention group and	83% were women	1095	1087	2182	Unguided, digital mindfulness-based self-help (app for practices and app	10 min per day	125 days	Not applicable	Only app for psychoeducation	Well-being was assess using Short Warwick Edinburgh Mental Well-being Scale	In intention-to-treat analysis, the intervention improved well-being but had no

					40.6 ± 11.0 for control group					for psychoeducation)					Emotional exhaustion, depersonalization, and personal accomplishment were measured using Maslach Burnout Inventory<br><br>Sickness absence was measured using a self-reported number of sickness absence days in past three months	effects on burnout (emotional exhaustion, depersonalization, and personal accomplishment) and sickness absence
Bielderman 2021 [100]	Netherlands	Nursing staff working in dementia special care units	Cluster RCT	3 and 9 months	Mean age 41.1 ± 12.2 years for intervention group and 40.3 ± 10.4 for control group	96.3% were women	168	129	297	An educational program to manage challenging behavior	A 3-day training course (each session 2–5 h) plus two 2.5 h follow-up sessions after three and six months	6 months	Not reported	No intervention	Burnout, job demands and job satisfaction<br><br>Burnout was measured using the 20-item Dutch version of the Maslach Burnout Index: the Utrecht Burnout Scale—C<br><br>Job satisfaction was assessed using the 6-item “Leiden Quality of Work Questionnaire for Nurses”<br><br>Job demands were assessed using the 5-item “Leiden Quality of Work Questionnaire”	The intervention had no effects on emotional exhaustion, depersonalization, personal accomplishment, job demands, and job satisfaction
Cascales-Perez 2021 [68]	Spain	Primary care health professionals	Individually RCT	8 weeks	Mean age 52.4 ± 9.4 years for intervention group and 49.6 ± 9.7 years for	81% were women	30	28	58	A mindfulness-based stress reduction intervention	A 2.5-h weekly session plus home exercises	8 weeks	Not reported	Received one 2.5-h theoretical training session	Burnout<br><br>The 22-item Maslach Burnout Index measured emotional exhaustion,	Overall burnout, emotional exhaustion, and depersonalisation were lower in intervention group than

					control group										depersonalization, and personal accomplishment	control group at 8 weeks post-intervention<br><br>Personal accomplishment did not differ between intervention and control group
Das Gecim 2021 [32]	Turkey	Nurses working at a university hospital	Individually RCT	3 months	45–59 years. Mean age 47.0 ± 3.7 years	Women	30	30	60	Intervention consisted of health education, yoga training and individualized nutrition counselling	One session per week for health education and an hour yoga session twice a week	4 weeks. Participants were encouraged to do yoga at home for 8 weeks	Workplace	No intervention	Work ability was measured using 7-item work ability index	Work ability index increased in intervention group at three months follow-up (difference 2.7 ± 3.5) but it did not change in control group (difference -0.07 ± 2.8). *p* value for between-group difference was 0.001
Fendel 2021 [64]	Germany	Resident physicians younger than 45 years	Individually RCT	2, 6 and 12 months	Mean age 31.0 ± 3.4 years	65.3% were women	76	71	147	Tailored mindfulness-based program	8 guided group sessions (one 135-min per week), one full day (6-h) silent retreat plus a 4-month maintenance phase (3 monthly booster sessions) and receiving weekly coursebooks	6 months	Evening sessions	Control group received weekly coursebook but without description of practical exercises	Burnout, perceived job strain and absenteeism<br><br>Burnout was measured using the 19-item Copenhagen Burnout Inventory<br><br>Perceived job strain was measured using the 8-item Irritation Scale<br><br>Absenteeism was measured by register data on the number of sickness absence days during 12 months of the study period	The level of burnout was lower in intervention group than control group at 6-month follow-up (mean difference −4.8, 95% CI −9.52, −0.11, $p = 0.046$), but there were no differences between the groups at 2- and 12-month follow-ups<br>The size of effect at 6-month was small (d = 0.32)<br><br>Perceived job strain was lower in intervention group than

															control only at 2-month follow-up but not at 6- or 12-month follow-ups The size of effect at 2-month was small (d = 0.29) Number of sickness absence days did not differ between intervention and control groups
Lebares 2021 [73]	USA	Resident physicians	Individually RCT	50 weeks for RCT 1 and 32 weeks for RCT 2	Mean age 27.4 ± 2.1 for intervention group and 28.6 ± 2.7 for control group in RCT 1 and 28.8 ± 2.4 for intervention group and 28.7 ± 2.2 for control group in RCT 2	50% of RCT 1 and 46.5% of RCT 2 were women	22 for RCT 1 and 22 for RCT 2	18 for RCT 1 and 21 for RCT 2	40 for RCT 1 and 43 for RCT 2	A tailored mindfulness-based cognitive training program	A 2-h weekly class in RCT 1 and a 1.5-h weekly class in RCT 2, weekend retreat hike after week 4, and 20-min daily skills practice outside class	8 weeks in RCT 1 and 6 weeks in RCT 2	Outside work hours	No intervention	The 9-item Maslach Burnout Inventory-Human Service Survey was used for measuring burnout in RCT 1 and a validated 2 items measure was used to assess emotional exhaustion and depersonalization in RCT 2	Burnout (emotional exhaustion and depersonalization) did not differ between intervention and control groups at 50-week follow-up in RCT 1<br><br>Emotional exhaustion (Cohen d = 0.81) and depersonalization (Cohen d = 0.69) were lower in intervention group than control group at 32-week follow-up in RCT 2
Montaner 2021 [87]	Spain	Healthcare professionals caring for dementia patients mostly nurses and nursing assistants	Individually RCT	3 and 12 months after intervention	19–64 years. Mean age 40.5 ± 12.8 for intervention group and 41.8 ± 12.3 for control group	93.3% were women	51	54	105	Acceptance and Commitment Therapy intervention to increase psychological flexibility	Six 1.5-h sessions	Not reported	The intervention was conducted in the work setting but outside of work hours	No intervention	The 22-item Maslach Burnout Index measured emotional exhaustion, depersonalization, and personal accomplishment	The intervention group had statistically significantly lower emotional exhaustion than control group at 3 months follow-up but not at 12 months

																The intervention had no effect on depersonalization<br><br>The intervention group had statistically significantly higher personal accomplishment than control group at both 3- and 12-month follow-ups
Pelit-Aksu 2021 [75]	Turkey	Student nurse interns working at gynecology and obstetrics clinics	Individually RCT	Post-intervention (3 weeks)	Mean age 21.92 ± 0.84 years for intervention group and 22.07 ± 0.86 year for control group	86.6% of intervention group and 83.3% of control group were women	67	78	145	Progressive muscle relaxation exercise	30 min per day using an audio for at least 4 days per week	3 weeks	Outside work	No intervention	Burnout<br><br>It was measured using the 10-item Burnout Measure Short version	Burnout was lower in intervention group than control group at post-intervention (*p* = 0.010)
Profit 2021 [81]	USA	Neonatal intensive care unit healthcare workers	Cluster RCT	1 month	Not reported	83% were women	100	233	333	Web-based enhancing resilience based on positive psychology	Six guided modules including educational material and practice-based learning	6 months	During evening	No intervention	Burnout and happiness<br><br>Emotional exhaustion was measured using five items of the Maslach Burnout Inventory<br><br>Subjective happiness was assessed using the subjective happiness scale	Intervention group had lower emotional exhaustion than control group (between-group difference − 5.3, 95% CI −10.4 to −0.1, *p* = 0.046)<br><br>Happiness did not differ between two groups
Sasaki 2021 [109]	Vietnam	Full-time registered nurses from a public tertiary hospital	Individually RCT	3 and 7 months	22–58 years. Mean age 33.1 years	84.9% were women	N = 317 for free order and N = 316 for fixed order	316	949	A smartphone stress management program based on cognitive behavioral therapy	A 15-min weekly session. One group received free choice (to complete a module each week in any order) and the second group received fixed order (to complete a module each week in a fixed order)	6 to 10 weeks	Not reported	No intervention	Work engagement<br><br>It was assessed using the 9-item short form of the Utrecht Work Engagement Scale	The intervention in a fixed order improved work engagement at three months (*p* = 0.049) but not at seven months follow-up However, the effect at 3-months

																was small (d = 0.16) and work engagement did not differ between intervention in a fixed order and control group when the 3- and 7-month data were combined<br><br>The intervention in free order did not improve work engagement
Smoktunowicz 2021 [104]	Sweden	Healthcare workers	Individually RCT	Post-intervention and 6 months after baseline	20–66 years. Mean age 36.2 ± 10.2 years	86.6% were women	N = 311 for self-efficacy + social support group and N = 311 for social support + self-efficacy group	N = 309 social support group and N = 309 self-efficacy group	1240<br><br>High attrition rate (82.5%) at follow-up	A self-guided internet intervention consisting of self-efficacy and perceived social support.<br>One intervention group received perceived social support and self-efficacy sequential enhancement module and the second intervention group received self-efficacy and perceived social support sequential enhancement module	One 1.5-h session per week.<br><br>Self-efficacy enhancement consisted of three exercises: mastery experience, vicarious experience, and action planning<br><br>Perceived social support enhancement consisted of three exercises: received support and cognitive distortions, social skills and peer support, and action planning<br><br>Every participant had access to four optional modules: relaxation exercises, mindfulness exercises, cognitive restructuring, and lifestyle (physical activity and pleasant activities)	6 weeks	Not reported	One control group received self-efficacy enhancement and the second control group received perceived social support enhancement, once a week for three weeks	Burnout and work engagement<br><br>Burnout was assessed by the 16-item Oldenburg Burnout Inventory<br>Work engagement was assessed by the 3-item Utrecht Work Engagement Scale-3	Burnout and work engagement did not differ between the intervention and control groups combing post-intervention and 6-month follow-up data<br>However, work engagement was significantly lower in the intervention group received self-efficacy and perceived social support sequential enhancement module than in the control group received perceived social support enhancement (d = −0.20; 95% CI −0.36 to −0.04)
West 2021 [89]	USA	Physicians	Individually RCT	6 and 12 months (post-intervention and 6 months	33.3% aged 40 or younger, 30.1% aged	43% were women	62	61	123	Self-facilitated discussion about well-being (experience,	One 1-h group discussion during a shared meal every two weeks	6 months	During a meal	No intervention	Burnout and job satisfaction	Using continuous outcomes, mean overall burnout, emotional

				after completion of the intervention)	41–50 years and 36.6% aged 51 or older					reflection, and small group learning)					Burnout was measured using the Maslach Burnout Index<br><br>Job satisfaction was assessed using the 5-item of Global Job Satisfaction domain of the Physician Job Satisfaction Scale	exhaustion, depersonalization, and personal accomplishment did not differ between intervention and control groups at 6- and 12-month follow-ups<br><br>Using dichotomized outcomes, high emotional exhaustion, high depersonalization, and low personal accomplishment significantly decreased in intervention group than control group at 6- and 12-month follow-ups, but overall burnout decreased in intervention group only at 12-month follow-up<br><br>Job satisfaction did not differ between intervention and control groups
Ameli 2020 [72]	USA	Healthcare professionals	Individually RCT	5 weeks	Median age (IQR) 28 (23–49) for intervention group and 34 (24–48) for control group	83% were women	43	35	78	A brief mindfulness-based intervention	A 1.5-h weekly session plus daily home practices	5 weeks	During work hours	No intervention	Burnout<br><br>Two items from the Maslach Burnout Inventory were used to assess emotional exhaustion and depersonalization	Emotional exhaustion and depersonalization did not differ between intervention and control groups

Becker 2017,[28] 2020 [29]	Germany	Registered nurses from five hospitals who had a musculoskeletal disorder at the shoulder or spine	Individually RCT	22 weeks and 24 months	24–61 years. Mean age 43.8 ± 10.3 years	86.8% were women	33	32	65	A 1.5-h psychoeducational session, five bi-weekly 1.5-h work-related psychosocial coaching (based on selection, optimization, and compensation model) and a weekly 45-min session of physiotherapy exercises	One psychosocial coaching every two weeks and one physiotherapy every week	10 weeks	Not reported	A weekly session (45 min) of physiotherapy exercises for 10 weeks	Work ability was assessed with three items from the German version of work ability index<br><br>Current work ability was assessed using one item of work ability index<br><br>Emotional exhaustion and depersonalization subscales of burnout were each measured using three items of the Maslach Burnout Inventory	The intervention improved work ability with respect to physical demands at 11 (d = 0.54) and 22 (d = 0.59) weeks after the start of intervention [28]<br><br>The intervention had no effects on current work ability and work ability with respect to psychological demands.[28]<br><br>The intervention reduced emotional exhaustion (d = 0.52) during the follow-up but had no effect on depersonalisation [28]<br><br>At 24 months follow-up among 24 participants in intervention group and 20 in control group, the intervention had no effect on work ability and well-being [29]
Chesak 2020 [99]	USA	Nurse leader mothers	Individually RCT	Post-intervention and 3-month follow-up	Not reported	Women only	18	18	36	An authentic connections groups program to enhance resilience	An hour weekly facilitated group discussion	12 weeks	During lunch break	An hour of free time per week	Burnout<br><br>Emotional exhaustion and personal accomplishment were measured using the Maslach Burnout Index	Emotional exhaustion and personal accomplishment did not differ between intervention and control groups at post-intervention

																and 3-month follow-up
Ghawadra 2020 [114]	Malaysia	Nurses with mild or moderate stress, Anxiety, or depression from a large teaching hospital	Cluster RCT	Post-intervention and 8 weeks post-intervention	38.4% aged 25 or younger, 32.6% aged 26–30 years and 29% aged 31 years or older	95.1% were women	118	106	224	A mindfulness-based stress reduction intervention	A 2-h workshop plus 4 weeks guided (by a website) self-practices	4 weeks	Home practises	No intervention	Job satisfaction<br><br>It was assessed using the 24-item Job Satisfaction Scale for Nurses	The intervention improved job satisfaction (p = 0.040), but the effect was small
Grabbe 2020 [50]	USA	Nurses from two tertiary care hospitals	Individually RCT	One week, three months, and one year after intervention	Mean age 45.3 years	95% were women	40	37	77	A community resiliency training (psychoeducation and sensory awareness skills)	A 3-h class plus access to the free app on community resiliency training	One class only	Workplace, outside work hours	A 3-h class on nutrition and healthy eating plus access to the free app on nutrition	Well-being and burnout<br><br>Well-being was assessed using the WHO-5 Well-being Index<br><br>Work-related burnout was measured with the 7-item Copenhagen Burnout Inventory	Well-being and burnout did not differ between intervention and control groups
Guo 2020 [105]	China	Registered nurses from a tertiary general hospital with burnout symptoms (the Maslach Burnout Inventory-General Survey score above 1.5)	Individually RCT	6 months	Mean age 27.8 ± 5.4 years for intervention group and 28.7 ± 5.1 for control group	98.6% were women	33	40	73	Three Good Things psychotherapy via WeChat.<br><br>Nurses in intervention group recorded three impressive things every day, and answered why these good things happened and what their role in those good things was	5 days per week	6 months	Not applicable	No intervention	Job performance was measured using a 16-item scale and consists of three subscales (job contribution, task performance and interpersonal support)	The intervention significantly improved job contribution, task performance and interpersonal support
Hilcove 2020 [62]	USA	Healthcare workers	Individually RCT	6 weeks	24–69 years. Mean age 42.4 ± 12.1 years	94.9% were women	41	37	78	Mindfulness-based yoga exercises	Six sessions plus home exercises 3–5 times per week	6 weeks	Home exercises	No intervention	Burnout<br><br>Burnout was measured using the 22-item Maslach Burnout Index	Burnout was lower in the intervention group compared with the control group (p = 0.003)

Leontjevas 2020 [122]	Netherlands	Care staff of nursing homes	Cluster RCT (a stepped-wedge design)	4, 12, and 20 months	Mean age 39.4 ± 11.9 years	96% were women	796	796	796	A multidisciplinary care program for depression consisting of procedures for screening, identification, diagnosis, treatment, and monitoring	A 3-h training workshop and then implementation	For five groups the program was implemented in one group at the time	Workplace	No intervention in control period	Job demands and job satisfaction<br><br>Job demands were assessed by 8-item scale<br><br>Job satisfaction was assessed by the 21-item Maastricht Work Satisfaction Scale for Healthcare	Job demands and job satisfaction did not differ between intervention and control groups
McGonagle 2020 [82]	USA	Community and hospital primary care physicians	Individually RCT	3 and 6 months	Mean age 43.4 ± 8.8 for intervention group and 41.8 ± 7.4 for control group	72.4% of intervention group and 86.2% of control group were women	29	30	59	A positive psychology coaching	One session every two weeks. First session lasted 60 min and five others lasted 30 min	3 months	Not reported	No intervention	Burnout, work engagement, and job satisfaction<br><br>The 22-item Maslach Burnout Index measured emotional exhaustion, depersonalization, and personal accomplishment<br><br>Work engagement was measured using a 17-item scale<br><br>Job satisfaction was assessed using three items	The intervention statistically significantly reduced burnout and improved work engagement and job satisfaction<br>The beneficial effects sustained up to six months follow-up
Pehlivan 2020 [102]	Turkey	Oncology–haematology nurses	Cluster RCT	Post-intervention 3-, 6-, and 12-month follow-ups	Mean age 25.0 ± 5.1 years for 2-day intervention 27.8 ± 5.3 years for 5-week intervention and 27.2 ± 5.3 years for control group	89.6% were women	N = 34 for 2-day intervention N = 49 for 5-week intervention	42	125	The compassion fatigue resiliency program	One group received 5 h per day for 2 days. The second group received 2 h per week for five weeks	5 weeks	Not reported	No intervention	Burnout<br><br>Burnout was measured using the 30-item professional quality of life scale-IV	Burnout did not differ between the groups

Ricou 2020 [98]	Switzerland	ICU nurses and nursing assistants	Individually RCT	Post-intervention and 6 months after intervention	70% aged 40 years or younger and 30% were older than 40 years	79% were women	51	60	111	A psychological intervention based on problem-based learning to boost personnel empowerment at the workplace	A weekly session	12 months	During work hours	No intervention	Burnout<br><br>The 22-item Maslach Burnout Index measured emotional exhaustion, depersonalization, and personal accomplishment	Overall burnout, emotional exhaustion, depersonalization, and personal accomplishment did not differ between intervention and control groups at post-intervention and 6-month follow-up
Sampson 2019 [112] and 2020 [111]	USA	Newly licensed registered nurses	Cluster RCT	Post-intervention, 3 and 6 months post-intervention	Mean age 24.3 years for intervention group and 24.5 years for control group	85.4% were women	47	42	89	A cognitive behavioral skill building program to improve physical and mental health	A 30–35 min weekly session	8 weeks	Workplace	A 30–35 min weekly discussion about challenging events experienced in the past week as part of the normal nurse residency program	Job satisfaction<br><br>It was measured using the 7-item Job Satisfaction Scale	Job satisfaction at 6 months follow-up was higher in the intervention group than control group (*p* = 0.025) [111]<br><br>However, job satisfaction did not differ between intervention and control group at post-intervention and three months follow-up[112]
Taylor 2020 [91]	Australia	Junior physicians	Individually RCT	8 weeks	23–37 years. Mean age 30 ± 4 years	76% were women	11	10	21	A personalised trauma-informed yoga intervention	An hour private yoga per week plus a 4-h workshop/retreat, audio-guided breathing and relaxation, and 2 eHealth video classes.<br><br>The participants were encouraged to do 2 h homework	8 weeks	Not reported	At least one 45–60 min group interval and/or Boxfit fitness per week.<br><br>The participants were encouraged to do 2.5 h homework	Burnout<br><br>Burnout was measured using the 22-item Maslach Burnout Inventory	Emotional exhaustion and personal accomplishment did not differ between the two groups, but depersonalisation was reduced more in yoga group than in fitness group (*p* = 0.05)<br><br>Emotional exhaustion did not significantly reduce in both groups, but personal

																accomplishment significantly increased in both groups
Wong 2020 [120]	Malaysia	Healthcare professional	Cluster RCT	17 months	Mean age 33.4 ± 7.7 years for intervention group and 33.3 ± 7.5 for control group	83% were women	544	498	1042	Supervised yoga	Sessions with handouts and home practice	8 weeks	Workplace	No intervention	Job satisfaction<br><br>It was measured using the 6-item scale	The intervention had no effect on overall job satisfaction
Xie 2020 [59]	China	ICU nurses with burnout	Cluster RCT	Post-intervention, 1 and 3 months post-intervention	Mean age 27.7 ± 7.7	Women only	45	46	91	Mindfulness-based intervention	A 2.5-h weekly course plus homework practices	8 weeks	Outside work hours	Two 1.5-h educational sessions in week 1 and week 4	Burnout<br><br>It was measured using the 22-item Maslach Burnout Inventory	Emotional exhaustion and depersonalization were significantly lower and personal accomplishment was significantly higher in intervention group than control group at 1- and 3-month follow-ups
Dunne 2019 [86]	Ireland	Emergency department healthcare professionals	Individually RCT	2 months (one week after completion of intervention)	Older than 18 years	Not reported	17	25	42	An attention-based training intervention	Four sessions (4 h/session) plus 20 min practice twice a day	7 weeks	Not reported	No intervention	Burnout<br><br>It was measured using the 22-item Maslach Burnout Inventory	Emotional exhaustion was lower in intervention group than control group, but depersonalization and personal accomplishment did not differ between intervention and control groups
Fukui 2019 [127]	Japan	Health and social care professionals (home care nurses, care managers, and heads of care workers)	Cluster RCT	7 months	Mean age 47.1 ± 8.6 years	95.3% were women	156	135	291	A multidisciplinary end-of-life educational intervention consisting of two educational workshops and an	Two 3-h programs	7 months	Not reported	No intervention	Job satisfaction<br><br>It was assessed using the 20-item short-form Minnesota Satisfaction Questionnaire	Job satisfaction did not differ between intervention and control groups

										educational booklet						
Kloos 2019 [49]	The Netherlands	Nursing home staff	Cluster RCT	12 weeks	16–65 years. Mean age 41.8 ± 12.1 years	95% were women	79	49	128	An online multi-component positive psychology intervention consisting of psychoeducation and positive psychology exercises	One module per week (eight modules)	8 to 12 weeks	A mandatory course with 9 h of work payment	No intervention	General well-being, work engagement and job satisfaction<br><br>General well-being was assessed by the Mental Health Continuum-Short Form<br><br>Work engagement was assessed by the 9-item short version of the Utrecht Work Engagement Scale<br><br>Job satisfaction was assessed using five items from the Maastricht Job Satisfaction Scale	The intervention had no effects on general well-being, work engagement and job satisfaction
Lebares 2019 [70]	USA	First-year surgery residents	Individually RCT	Post-intervention (3.5 months after baseline), and 1-year after baseline	Mean age 29.0 ± 2.4 years for intervention group and 27.4 ± 2.1 for control group	38.1% were women	12	9	21	A mindfulness-based cognitive training	A 2-h weekly session	8 weeks	Not reported	No intervention	Burnout<br><br>It was measured using the 12-item abbreviated Maslach Burnout Inventory	Burnout did not differ between intervention and control groups at post-intervention and 1-year follow-up
Lin 2019 [118]	China	Nurses from two tertiary-level general hospitals	Individually RCT	Post-intervention and 3 months follow-up	Mean age 32.9 ± 7.5 years for intervention group and 30.2 ± 6.1 years for control group	93.3% were women	44	46	90	A modified mindfulness-based stress reduction intervention	A 2-h weekly session plus 20-min daily home practices for 6 days of a week	8 weeks	Not reported	No intervention	Job satisfaction<br><br>Nurses' job satisfaction was assessed using the 31-item McCloskey/Mueller Satisfaction Scale	Job satisfaction did not differ between intervention and control group at post-intervention and 3-month follow-up
O'Brien 2019 [132]	USA	Nurses and nursing aides	Individually RCT	One month after completion of intervention	Mean age 37.9 ± 13.2 years	86% were women	37	34	71	Acceptance and commitment therapy	A 2.5-h weekly session	2 weeks	Not reported	No intervention	Work-based injuries and sickness absence due to injury	Intervention group reported fewer days missed from work due to injury than control group (*p* =

														Self-reported information on number of days missed from work due to work related injuries in the past month<br><br>Self-reported information on frequency of work-related strains and muscle pulls, bites, scratches, wounds, cuts, and bruising in the past month	0.008) at 1-month follow-up<br><br>Frequency of work-related injuries did not differ between intervention and control group
Soler-Font 2019 [136]	Spain	Nurses and nursing aides from two third-level hospitals	Cluster RCT	6 and 12 months	31–49 years. 16.0% aged 30 years or younger, 52.9 aged 31–49 years and 31.1% aged 50 years or older	84.1% were women	138	119	257	A multifaceted intervention consisting of participatory ergonomics, healthy lifestyle promotion, and a tailored case management	Participatory ergonomics consisted of diagnostic, treatment, and implementation.<br><br>Healthy lifestyle promotion consisted of Nordic walking (a 1.5 h weekly session for 12 weeks), a web platform for healthy diet and mindfulness training (a 2-h weekly session for four weeks).<br><br>Case management lasted for 10 months	12 months	Not reported	No intervention	Sickness absence<br><br>Registered data on the number of sickness absence days and spells due to musculoskeletal disorders	Sickness absence did not differ between intervention and control groups
Watanabe 2019 [71]	Japan	Nurses from four general hospitals	Individually RCT	13, 26 and 52 weeks	21–55 years. Mean age 30.1 ± 8.4	Women only	40	40	80	A mindfulness-based stress management intervention	A 30-min weekly session	4 weeks	Not reported	Received a psychoeducation leaflet	Burnout<br><br>It was measured using the 22-item Maslach Burnout Inventory	Emotional exhaustion, depersonalization and personal achievement did not differ between intervention and control groups at 13-, 26- and 52-week follow-ups
Khamali 2018 [52]	France	ICU nurses	Individually RCT	6 and 12 months	48% aged 30 years or younger, 44.4% aged 31–40 years	58% were women	101	97	198	A course on role-playing/simulation, and discussion of practices to cope with work	A 5-day course (3 consecutive days in the 1st week and 2 consecutive days in the 2nd week)	5 days	During work hours	No intervention	Job strain, psychological demand, decision latitude, social support, self-rated	Prevalence of job strain (high psychological demand and low decision latitude)

and 7.6%<br>aged 41<br>years or<br>older	stressors and<br>stressful situations<br>at workplace	health, and job satisfaction<br><br>Work-related job strain was measured using the 26-item Job Content Questionnaire<br><br>Burnout at work and general health was assessed using some items from the 46-item Copenhagen Psychosocial Questionnaire<br><br>Job satisfaction was assessed using a single item scale (0 to 10)	at 6-month follow-up was lower in intervention group than control group (13% vs. 67%, $p <$ 0.001). The effect sustained at 12 months follow-up (37% vs. 72%, $p <$ 0.001)<br><br>Prevalence of isostrain (job strain and low social support) at 6-month follow-up was lower in intervention group than control group (7% vs. 55%, $p <$ 0.001). The effect sustained at 12 months follow-up (14% vs. 67%, $p <$ 0.001)<br><br>Decrease in psychological demand at 6 months was larger in intervention group than control group (−5.5 vs. −0.7, $p <$ 0.001)<br><br>Increase in decision latitude at 6 months was larger in intervention group than control group (18.4 vs. −5.1, $p <$ 0.001)

																Increase in social support at 6 months was larger in intervention group than control group (2.0 vs. −2.6, $p < 0.001$)<br><br>Symptoms of burnout at 6-month follow-up were less common in intervention group than control group (87.4 vs. 51.2, $p = 0.001$)<br><br>Job satisfaction at 6 months was higher in intervention group than control group (82.5 vs. 54.9, $p < 0.001$)<br><br>Self-rated general health at 6 months was higher in intervention group than control group (88.8 vs. 80.2, $p = 0.01$)
Maatouk 2018 [37]	Germany	Nurses aged 45 years or older	Individually RCT	After booster session (13 weeks after baseline assessment)	45 years or older. Mean age 51.6 ± 4.7 for intervention group and 52.6 ± 5.6 for control group	87% were women	52	55	107	A small-group intervention for the promotion of mental health according to a theory of successful ageing	A 2-h weekly session and a booster session 6 weeks after completion of the first 7 sessions	7 weeks	During work time	No intervention	Well-being, work ability, and job control<br><br>Well-being was measured using the 5-item WHO questionnaire	Well-being, work ability, and job control did not differ between intervention and control groups at follow-up

															Work ability was measured using three items from Work Ability Index<br><br>Job control was assessed using the 9-item work analysis instrument for hospitals-self report version	
Mache 2018 [79]	Germany	Junior physicians working in departments of emergency medicine	Individually RCT	Post-intervention, 3- and 6-month follow-up	Mean age 27.3 ± 2.5 years for intervention group and 27.1 ± 2.1 years for control group	63% were women	35	35	70	Coping skill training consisting of problem solving and emotion regulation strategies	A 1.5-h weekly session	3 months	Not reported	No intervention	Emotional exhaustion, job satisfaction and work engagement<br><br>Emotional exhaustion was measured using the 5-item Maslach Burnout Inventory<br><br>Job satisfaction was assessed with the job satisfaction scale of the Copenhagen Psychosocial Questionnaire<br><br>Work engagement was measured using the Utrecht Work Engagement Scale	Emotional exhaustion was lower in intervention group than control group at post-intervention (d = 0.61), 3-month (d = 0.50) and 6-month follow-ups ($p <$ 0.01, all)<br><br>Job satisfaction (d = 0.35) and work engagement (d = 0.22) were higher in intervention group than control group at post-intervention ($p < 0.1$), but they did not differ between intervention and control groups at 3- and 6-month follow-ups
Mistretta 2018 [48]	USA	Employees of a tertiary medical centre	Cluster RCT	6 weeks and 3 months	22–80 years. Mean age 46.0 ± 12.6	86.7% were women	N = 22 for in-person mindfulness -based group and N = 23 for smartphone-	15	60	In-person mindfulness resilience training, or smartphone-delivered resilience training	For in-person group, a 2-h weekly session.<br><br>Smartphones group focused on one of 4 topics every 7–10 days (sleep,	6 weeks	During work time	No intervention	Emotional well-being and Burnout<br><br>Emotional well-being was assessed using the 5-item WHO questionnaire	Emotional well-being improved and emotional exhaustion reduced between baseline and 3 months follow-up in in-person

							delivered group				happiness/positivity, energy/focus, and productivity)				Burnout was measured by Maslach Burnout Inventory	mindfulness resilience training group. The intervention did not improve depersonalization and personal accomplishment subscales of burnout<br><br>Only well-being improved in smartphone group<br><br>However, emotional well-being and emotional exhaustion did not differ between in-person mindfulness, smartphone, and control groups
Schroeder 2018 [60]	USA	Primary care physicians	Individually RCT	Post-intervention and 3 months	32–61 years. Mean age 42.8 ± 8.4 years	73% were women	17	16	33	A brief mindfulness-based intervention	13-h training, 2-h follow-up sessions 2 and 4 weeks after training	4 weeks	Weekend	No intervention	Burnout<br><br>It was measured using the 22-item Maslach Burnout Inventory	The intervention reduced emotional exhaustion and depersonalization at 3 months follow-up
Suni 2018 [130]	Finland	Healthcare workers with recurrent non-specific low back pain	Individually RCT	6 and 12 months	30–55 years, mean age 46.4 ± 6.8 years	Women	Exercise group (N = 57), counselling group (N = 55) and both exercise and counselling group (N = 53)	54	219	Neuromuscular exercise (to enhance stability of the spine) and/or back care counselling	An hour exercise session, twice a week (a total of 48 sessions)<br><br>A 45-min session of counselling, once a week in 1st month and every third week after that (a total of 10 sessions)	24 weeks	On weekdays near the workplaces	No intervention	Sickness absence days	Mean sickness absence days during the first 6 months was 0.86 for exercise group, 0.97 for counselling group, 0.13 for combined exercise and counselling, and 1.56 days for control group. The differences between groups

																were not significant (p = 0.60)<br><br>Mean sickness absence days for 12 months follow-up was 4.17 days for exercise group, 2.30 for counselling group, 0.15 for combined exercise and counselling group, and 2.29 days for control group. The differences between groups were significant (p = 0.025)
Verweij 2018 [66]	The Netherlands	Resident physicians	Individually RCT	3 months	Mean age 31.2 ± 4.6 years	88% were women	71	67	138	Mindfulness-based stress reduction including psychoeducation and exercises (body scan, yoga, and sitting/walking).<br><br>They were instructed to practice 45 min per day at home	One 2.5-h session per week and one 6-h silent day	8 weeks	Sessions in the evening and silent day during the weekend	No intervention	Burnout<br><br>Emotional exhaustion and depersonalization subscales of burnout were each measured using three items of the Maslach Burnout Inventory	Reduced personal accomplishment was lower in the intervention group than control group at follow-up (−1.19 (−0.13 to −2.25, p = 0.028).<br><br>Emotional exhaustion (between-group mean difference 0.09, 95% CI −1.73 to 1.92, p = 0.92) and depersonalization (between-group mean difference 0.17, 95% CI −0.74 to 1.08, p = 0.71) did not differ between two groups
Calisi 2017 [47]	USA	Registered cardiac	Individually RCT	8 weeks	27–60 years	Women	24	22	46	Relaxation response, a	A 45-min session. Intervention group was	8 weeks	Workplace	No intervention	Well-being	Post-intervention well-being did

		nurses								diaphragmatic breathing practice, and a repetitive mental focus	encouraged to practice for 10–20 min, twice a day				It was measured with single item (score 0 to 7)	not differ between intervention and control groups
Ireland 2017 [65]	Australia	Intern doctors working at emergency department of in a hospital	Individually RCT	5 and 10 weeks	22–48 years. mean age 26.9 ± 4.8	64% were women	23	21	44	Mindfulness program including education and exercises	1-h weekly training workshop	10 weeks	Workplace	An hour extra break per week	Burnout<br><br>Burnout was measured with the 19-item Copenhagen Burnout Inventory	Burnout marginally improved in the intervention group (*p* = 0.072)
Mache 2017 [78]	Germany	Junior physicians working in Gynecology and Obstetrics Medicine of seven hospitals	Individually RCT	Post-intervention, 3- and 6-month follow-up	Mean age 27.1 ± 2.1 years for intervention group and 27.8 ± 2.2 years for control grou	69.2% were women	38	40	78	Coping skill training consisting of problem solving and emotion regulation strategies	A 1.5-h weekly session	3 months	During work hours	No intervention	Emotional exhaustion, and Job satisfaction<br><br>Emotional exhaustion was measured using the 5-item Maslach Burnout Inventory<br><br>Job satisfaction was assessed with the job satisfaction scale of the Copenhagen Psychosocial Questionnaire	Emotional exhaustion was lower in intervention group than control group at post-intervention, 3- and 6-month follow-ups (*p* < 0.01, all)<br><br>Job satisfaction was higher in intervention group than control group at post-intervention (*p* = 0.01), 3-month (*p* = 0.03) but not at 6-month follow-up (*p* = 0.3)
van Dijk 2017 [43]	Netherlands	First-year clinical clerkship medical students	Cluster RCT	3, 7, 12, 15, and 20 months	Median age and SD 23.7 ± 1.91 for intervention group and 23.3 ± 1.77 for control group	72% of intervention group and 85% of control group were women	83	84	167	A mindfulness-based stress reduction intervention	A 2-h weekly session plus home practices	8 weeks	Outside work hour	No intervention	Mental well-being.<br><br>Emotional, psychological, and social well-being was assessed using the 14-item Mental Health Continuum-Short Form	The intervention improved mental well-being (*p* = 0.002, Cohen's d = 0.44) during the 20-month follow-up
Wei 2017 [85]	China	Emergency departments nurses of 3 hospitals	Individually RCT	6 months	20–48 years. 80% were younger than 35 years.	86.3% were women	51	51	102	Regular and comprehensive management.	A 30-min session twice a week for regular management	6 months	Workplace	Regular management consisted of discussions about stressful	Burnout<br><br>A job burnout was measured by the 15-item revised	The intervention statistically significantly reduced emotional

										Regular management included discussions about stressful problems and solutions.<br><br>Comprehensive management included classes for improving communication skills, efficacy, emotional control, working skills, and conflict management				problems and solutions	Maslach Burnout Inventory–General Survey	exhaustion and depersonalization but had no effect on personal achievement
Bernburg 2016 [108]	Germany	Junior pediatricians from 10 hospitals	Individually RCT	6 months	Mean age 27 ± 2.1 years	70% were women	26	28	54	A psychosocial competence training consisted of psychosocial skills training (mindfulness, self-awareness, resilience), and cognitive-behavioral and solution-focused counselling	One 1.5 h session per week. Participants were instructed to do 20-min practices 5 times a week	12 weeks	Not reported	No intervention	Work engagement and job satisfaction<br><br>Work engagement was measured by the short version of the Utrecht Work Engagement Scale<br><br>Job Satisfaction was measured with 4 items	Job satisfaction significantly improved in the intervention group both at 3 and 6 months but no changes were found among the control group<br><br>The intervention had no effect on work engagement
Gill 2016 [126]	Vietnam	Community physicians' assistants	Individually RCT	6 months	Mean age 37.65 years for control group, 38.21 years for the 1st intervention group and 37.63 years for the 2nd intervention group	67.9% of control group, 70.6% of the 1st intervention group and 67.9% of the 2nd intervention group were women	N = 214 the 1st group and N = 212 the 2nd group	212	638	A phone-based text message intervention to improve medical knowledge	One group received a daily text message about primary care medicine, and they were asked to acknowledge receipt of the message.<br><br>The second group received a daily message about primary care medicine but formatted as a 4-option multiple choice question. They were asked to answer the question	6 months	Not reported	Received a weekly non-medical test message	Job satisfaction<br><br>It was assessed using the Brief Index of Affective Job Satisfaction	Job satisfaction did not differ between the groups at 6 months follow-up

Hersch 2016 [116]	USA	Nurses from six hospitals	Individually RCT	3 months	22–65 years. Mean age 41 years	87.5% were women	52	52	104	A web-based stress management intervention consisting of 7 modules for nurses and an additional module for nurse managers	To use as often as possible	3 months	Access the program on work time or at home	No intervention	Job satisfaction and workload<br><br>It was measured using the 24-item Nurse Satisfaction Scale<br><br>A subscale of Nursing Stress Scale was used to assess workload	Workload reduced in intervention group compared with control group (p = 0.022)<br><br>Job satisfaction did not differ between intervention and control groups
Kubota 2016 [93]	Japan	Oncology nurses	Individually RCT	3 months	Mean age 38.9 ± 5.4 years for intervention group and 40.0 ± 5.4 years for control group	96% were women	50	46	96	A psycho-oncology training consisting of supportive communication, and a crisis intervention approach to address psychological problems in cancer patients	Two 8-h meetings with one week interval	2 weeks	Not reported	No intervention	Burnout<br><br>Emotional exhaustion, depersonalization, and personal accomplishment were measured using the 22-item Maslach Burnout Inventory	Emotional exhaustion, depersonalization, and personal accomplishment did not differ between intervention and control groups
Rasmussen 2016 [33]	Denmark	Elderly care personnel (89% were nurses' aides and 11% service workers)	Cluster RCT (a stepped-wedge design)	12 weeks	18–65 years. Mean age 47 years	93% were women	586	586	586	A multi-faceted intervention consisting of integrated physical training, participatory ergonomics, and cognitive behavioural training	12 sessions of physical training, 5 sessions of participatory ergonomics, and 2 sessions of cognitive behavioural training	12 weeks	During work hours	No intervention in control period	Current work ability, sickness absence, physical exertion, and occupational lifting<br><br>Current work ability was assessed using one item of work ability index<br><br>Self-reported sickness absence days due to low back pain<br><br>Physical exertion was measured using a single item of Borg's Cr10-scale	Intervention reduced occupational lifting (difference −0.35, 95% CI −0.61 to −0.08, p = 0.01) and marginally improved work ability (difference −0.24, 95% CI −0.49 to 0.01, p = 0.06)<br><br>The intervention had no effects on sickness absence, and physical exertion

															Occupational lifting was assessed using a single item	
Ripp 2016 [95]	USA	First-year internal medicine residents	Individually RCT	12 months	Not reported	Not reported	21	17	38	Facilitated group discussion intervention	Two 1-h sessions monthly (total 18 sessions)	9 months	Workplace	No intervention	Burnout<br><br>It was measured using the Maslach Burnout Inventory	Burnout at follow-up did not differ between intervention and control groups
Steinberg 2016 [107]	USA	Surgical intensive care unit personnel	Individually RCT	1 week after completion of intervention (2 months after baseline assessment)	Mean age 44.0 ± 12.1 for intervention group and 44.1 ± 10.9 for control group	88% were women	16 ?	16 ?	32	Mindfulness-based intervention consisting of meditation, light yoga, and music	An hour per week	8 weeks	During work hours	No intervention	Work engagement<br><br>Work engagement was measured using the 9-item Utrecht Work Engagement Scale	Work engagement increased during the follow-up in intervention group (p = 0.006) but did not change in control group (p = 0.30)
Alexander 2015 [84]	USA	Nurses	Individually RCT	8 weeks	Mean age 46.4 ± 10.2	97.5% were women	20	20	40	Supervised yoga	Sessions with handouts and home practices	8 weeks	Workplace	No intervention	Burnout<br><br>Emotional exhaustion, depersonalization, and personal accomplishment were measured using the 22-item Maslach Burnout Inventor	Emotional exhaustion and depersonalization statistically significantly improved in intervention group than control group<br><br>Yoga had no effect on personal accomplishment
Amutio 2015 [67]	Spain	Physicians	Individually RCT	8 weeks and 12 months	Mean age 47.3 ± 9.4	57.1% were women	21	21	42	A mindfulness-based stress reduction intervention	A 2.5-h weekly session plus one 8-h retreat session. The intervention group was instructed to practice exercises for 45 min per day.<br><br>4 weeks after end of intervention phase, a 2.5-h monthly session continued for 10 months	8 weeks	Outside work	No intervention	Burnout<br><br>Emotional exhaustion, depersonalization, and personal accomplishment were measured using the 22-item Maslach Burnout Inventory	The intervention reduced overall burnout (p = 0.02) and emotional exhaustion (p = 0.05) at 8-weeks post-intervention but had no effects on depersonalization and personal accomplishment<br><br>The intervention effect on emotional exhaustion

															sustained at 12 months follow-up
Andersen 2015 [31]	Denmark	Healthcare workers with a history of work-related back or upper body musculoskeletal problems	Individually RCT	3 months	Mean age 45.1 years for intervention group and 47.1 years for control group	94.4% were women	27	27	54	Tailored aerobic fitness and strength exercises	A 1.5 h health education and 50 min exercises 3 times per week	10 weeks	During work hours	A 1.5 h health education	Current work ability and sickness absence<br><br>Self-reported number of sickness absence days due to musculoskeletal disorders<br><br>Current work ability was assessed using one item of work ability index	Current work ability improved in intervention group compared with control group ($p = 0.04$) Sickness absence days during the past three months did not differ between intervention and control groups
Jakobsen 2015 [27,40]	Denmark	Healthcare workers from 18 departments of three hospitals	Cluster RCT	10 weeks	18–67 years. Mean age 44 ± 10 for control group and 40 ± 12 for intervention group	Women	111	89	200	Supervised high-intensity strength exercises plus five group coaching for 30–45 min to motivate workers to take part in the training and to begin and maintain healthy lifestyles.<br>They were also offered courses (each 1.5–3 h) on ergonomics, patient handling and use of assistive devices	10 min 5 times per week	10 weeks	Workplace	They received training equipment and posters demonstrating exercises and performed exercises at home.<br>They were also offered courses (each 1.5–3 h) on ergonomics, patient handling and use of assistive devices	Change in work ability index measured with 7 items [27] and perceived change in well-being and job satisfaction during 10-week follow-up [40]	During follow-up, work ability index did not change in the intervention group but decreased in the control group [27]. Work ability was higher in the intervention group compared with the control group (Cohen's d 0.24, 95% CI 0.20, 0.50) [27]<br><br>51% of intervention group and 7% of control group reported improvement in well-being ($p < 0.001$) and 32% of intervention group and 5% of control group reported improvement in job satisfaction ($p < 0.001$) [40]

Linzer 2015 [74]	USA	Primary care clinicians	Cluster RCT	12–18 months	Mean age 48.3 ± 8.9 for intervention group and 46.4 ± 9.4 for control group	51.8% were women	83	83	166	Improvements in work conditions (communication, workflow, and quality)	Through a variety of methods during follow-up period	During follow-up	Workplace	No intervention	Burnout and Job satisfaction<br><br>It was measured with a 5-item scale, focusing primarily on emotional exhaustion and burnout was defined as a score of 3 or higher.<br><br>Job satisfaction was assessed with a questionnaire	During follow-up, burnout improved in 21.8% of intervention group and in 7.1% of control group ($p = 0.01$)<br><br>During follow-up, job satisfaction increased in 23.1% of intervention group and in 10.0% of control group ($p = 0.04$)
Mache 2015 [121]	Germany	Medical specialists in their first year after graduation	Individually RCT	3 and 6 months	Mean age 28 years	60% were women	42	43	85	A psychosocial intervention consisting of resilience training, and cognitive behavioral and solution-focused counselling	A 2-h weekly session	12 weeks	Workplace	No intervention	Job satisfaction<br><br>Job satisfaction was assessed using the 4-item of the Copenhagen Psychosocial Questionnaire	Job satisfaction did not differ between intervention and control groups
Müller 2015 [36]	Germany	Registered nurses of a community hospital	Individually RCT	12 months?	Mean age 44.7 ± 9.3 years for intervention group and 42.7 ± 9.9 for control group	94% were women	36	34	70	Training based on a life-management strategy of selection, optimization, and compensation model to cope with job demand or to increase job resources	6 sessions (16.5 h)	9 months	Workplace	No intervention	Work ability, well-being, and job control<br><br>Mental well-being was assessed with WHO well-being index perceived work ability was assessed with three items of the German version of work ability index, and job control was assessed by a 9-item scale	In intention-to-treat analysis, the intervention improved mental well-being ($p = 0.046$) but did not improve job control or perceived work ability<br><br>The intervention was effective in improving mental well-being only in those with low job control at baseline ($p < 0.001$) but not in those with high job control
Roussel 2015 [55]	Belgium	Hospital healthcare workers with	Individually RCT	6 months	18–65 years. Mean age 41.4 years	82.6% were women	31	38	69	A multidisciplinary program	10 group sessions and 5 individual sessions.	3 months	During work hours	No intervention	General health, job satisfaction, and sickness absence	General health, job satisfaction, and sickness

		physically demanding jobs			for intervention group and 40.4 for control group					consisting of (1) change in hospital policy (e.g., changes in work schedules, equipment, methods), (2) exercise and nutrition interventions, (3) ergonomics changes and (4) psychological intervention	Four 1-h exercise sessions, and one nutrition session and one cardiovascular session.<br><br>Six weekly sessions for ergonomics /2 individual and 4 group sessions).<br><br>3 1-h individual sessions for psychological intervention				Self-reported data on sickness absence due to musculoskeletal problems during the follow-up<br><br>General health was assessed by the 36-item short form health survey<br><br>Job satisfaction was assessed using the Psychosocial Aspects of Work	absence days did not differ between intervention and control groups
von Thiele Schwarz 2015 [39]	Sweden	Healthcare workers (majority were nurses or assistant nurses)	Cluster RCT	12 and 24 months	Mean age 46.7 ± 9.2 for intervention group and 45.0 ± 12.1 for control grou	96.4% of intervention group and 91.2% of control group were women	111	91	202	Integration of health protection and health promotion into continuous improvement system (Kaizen)	1 to 4 short meetings per month at the unit level for continuous identification of risks for Kaizen, 1-h weekly exercise session plus lectures on healthy lifestyles for health promotion, and a monthly meeting at the unit level and annual safety inspections for health protection.<br><br>Integration was implemented through multiple workshops and over 20 coaching meetings	24 months	During work hours	Health promotion and health protection were not integrated into Kaizen. Only annual safety inspection for health protection was integrated into Kaizen	Work ability, self-rated health, and sickness absence<br><br>Current workability and self-rated health were assessed using a single item<br><br>Information on frequency and duration of sickness absence during the past 12 months was collected using self-reports	Current work ability, self-rated health, and frequency and duration of sickness absence did not differ between intervention and control groups
West 2014 [88]	USA	Practicing physicians	Individually RCT	9-month intervention period, and 3- and 12-month post intervention follow-ups	Not reported	33.8% were women	37	37	74	A facilitated small-group curriculum consisting of mindfulness, reflection, shared experience, and learning	1-h session every two weeks (a total of 19 sessions)	9 months	During work time	No intervention	Work engagement, burnout, and Job satisfaction<br><br>Burnout was measured using the Maslach Burnout Inventory<br><br>Work engagement was measured by the 12-item	Intervention improved work engagement at 3-month (p = 0.04) and 12-month (p = 0.03) post intervention<br><br>Intervention reduced high depersonalization at 3-month (p = 0.004) and 12

															Empowerment at Work Scale	months (p = 0.02) post intervention
															Job satisfaction was measured using the 12-item Physician Job Satisfaction Scale	Emotional exhaustion, overall burnout, and job satisfaction did not differ between intervention and control groups
Christensen 2013 [38]	Denmark	Healthcare workers (majority were Overweight)	Cluster RCT	3 and 12 months	Mean age 44.8 ± 9.5 for intervention group and 46.4 ± 9.5 for control group	96.5% were women	76	68	144	Intervention consisting of diet, strengthening and fitness exercises, and cognitive behavioral training	An hour per week	12 months	During work time	Lectures about healthy lifestyle	Work ability, and sickness absence	Work ability, number of days of short sickness absence and number of spells of short sickness absence did not differ between intervention and control groups at 3- or 12-month follow-up
Jeon 2013 [123]	Australia	Care workers of aged care homes (76% were unlicensed assistants in nursing or personal care assistants)	Cluster RCT	9 and 18 months from the baseline (the 2nd follow-up was conducted six months after completion of the intervention)	Mean age at baseline 46.5 years for intervention group and 47.1 years for control group	Not reported	202 at baseline, 229 at follow-up 1 and 240 at follow-up 2	301 at baseline, 315 at follow-up 1 and 342 at follow-up 2	503 at baseline, 544 at follow-up 1 and 582 at follow-up 2	A leadership and management program (an educational and support program for middle managers)	A workshop, individual meetings/teleconferences every 4–6 weeks, coaching/mentoring, and peer support meetings	12 months	Workplace	No intervention	Job satisfaction and injuries<br><br>Job satisfaction was assessed using a single item (a 10-point Likert scale) from the Workforce Dynamics Questionnaire<br><br>Fall with injury was assessed using register data during the follow-up	Job satisfaction and fall with injury did not differ between intervention and control groups
Kontio 2011 [151] and 2013 [124]	Finland	Nurses	Cluster RCT	3 (post-intervention) and 9 months	24–64 years. Mean age 43 ± 9 for intervention group and 45 ± 10 years for control group	54% were women	95	63	158	'ePsychNurse.Net' eLearning course consisting of six main modules.<br><br>Each module includes reading material, peer discussion,	120 h	3 months	One-third work hours and two-third nurses' own time	No intervention	Job satisfaction<br><br>Job satisfaction was measured using the 14-item Job Satisfaction Scale	Job satisfaction did not differ between intervention and control groups at 3- and 9-month follow-ups

										reflective journal, and individual exercise and assignment						
van de Ven 2013 [119]	The Netherlands	Nursing staff of care homes for people with dementia	Cluster RCT	4 and 8 months	Mean age 43.0 ± 10.9 years	98.4% were women	141	178	319	Dementia-care mapping, a multicomponent person-centred intervention to improve the quality and effectiveness of care	Training two mappers for each care home, running dementia-care mapping briefing day for organization and carrying out observation, feedback, and action plans twice for a period of two months each with an interval of four months	4 months	Workplace	No intervention	Job satisfaction<br><br>Job satisfaction was measured using the Maastricht Job Satisfaction Scale for Healthcare	Job satisfaction did not differ between intervention and control groups
Flannery 2012 [30]	USA	Minority nursing assistants working at long-term care facilities	Cluster RCT	6 months	Mean age 41.8 ± 13.0 years	Women	24	15	39	A 2-h session to assess work factors affecting health, a 30-min educational lecture to encourage participants to increase physical activity and reduce fat and salt consumption, and ongoing motivation for 3 months	Weekly	12 weeks	During work time	A 30-min education session on physical activity and diet	Work ability, effort reward, and Job satisfaction<br><br>Job satisfaction was assessed using the 21-item nursing home administrator job satisfaction scale<br><br>The effort reward and imbalance were assessed using the 16-item questionnaire<br><br>Work ability index was measured using the 7-item questionnaire	Work ability index increased in intervention group and decreased in control group during follow-up. The difference between the group was significant (*p* = 0.049)<br><br>Intervention had no effects on effort, reward, effort/reward ratio and job satisfaction
van der Molen 2011 [152] and 2012 [139]	The Netherlands	Healthcare workers of one academic hospital	Cluster RCT	6 and 12 months	Mean age 36 ± 12.8 years for needle safety device plus workshop group, 41 ± 11.7 years for workshop group and 38 ± 11.7	78% of needle safety device plus workshop group, 83% of workshop group and 75% of control group were women	267 for needle safety device plus workshop and 263 for workshop only	266	796	Needle safety device plus an hour workshop, or workshop only	One time only	An hour workshop	Workplace	No intervention	The number of self-reported and registered needle stick injuries in the past 6 months<br><br>Injuries were assessed using questionnaires and hospital notification registry	The proportion of workers with self-reported needle stick injuries was lower in needle safety device plus workshop group (OR 0.34, 95% CI 0.13–0.91) and workshop only (OR 0.45, 95% CI 0.19–1.06)

					years for control group											compared with control group<br><br>However, the rate of registered injuries did not differ between the three groups at 6- or 12-month follow-up
Brinkborg 2011 [58]	Sweden	Social workers	Individually RCT	10 weeks	24–64 years. Mean age 44.0 ± 11.1 years	89% were women	70	36	106	A brief stress management intervention to increase psychological flexibility	One 3-h session every two weeks plus physical exercise and mindfulness practice at home	8 weeks	Not reported	No intervention	Burnout, and perceived psychological demands and control<br><br>Emotional exhaustion, depersonalization, and personal accomplishment were measured using the 22-item Maslach Burnout Inventory<br><br>Work-related demand, control and support were assessed using the Demand -Control -Support Questionnaire	The intervention reduced overall burnout, emotional exhaustion, and depersonalization, and increased personal accomplishments<br><br>The intervention had no effects on perceived psychological demands and control
Redhead 2011 [90]	UK	Nurses working in a low-secure mental health unit	Individually RCT	8 months	Mean age 39.5 ± 2.2	60% were women	12 qualified and 10 unqualified nurse	9 qualified and 11 unqualified nurses	42	Psychosocial intervention (cognitive behaviour therapy and coping strategy enhancement)	16 half-day sessions for qualified nurses and 8 half-day sessions for unqualified nurses	8 months	Workplace	No intervention	Burnout<br><br>Burnout was measured with the Maslach Burnout Inventory	Intervention statistically significantly reduced depersonalization but not emotional exhaustion and personal achievement among qualified nurses<br><br>Intervention did not reduce burnout among

																unqualified nurses
Günüsen 2010 [97]	Turkey	Nurses	Individually RCT	Post-intervention and 6 months follow-up	Most aged 19–34 years	Women only	N = 30 coping training and N = 31 social support	28	89	(1) Coping training consisting of cognitive and the problem-solving coping strategies.<br><br>(2) Social support	A 1.5–2 h weekly session	7 weeks	During work hour	No intervention	Burnout<br><br>Burnout was measured with the Maslach Burnout Inventory	Emotional exhaustion, depersonalization and personal accomplishment did not differ between the groups
Bygren 2009 [51]	Sweden	Medical care staff	Individually RCT	8 weeks	Mean age 49.8 for intervention group and 50.4 for control group	93.1% were women	51	50	101	Cultural participation (films, concerts, art exhibitions, or choir singing session)	One session per week	8 weeks	Outside work	No intervention	Perceived general health<br><br>It was assessed using the short form health questionnaire (SF-36)	The intervention improved physical health (difference 3.55 points, 7.5%)
Fillion 2009 [117]	Canada	Palliative care nurses	Individually RCT	Post-intervention and three months follow-up	Mean age 44.1 ± 10.6 years	99.1% were women	56	53	109	A meaning-centered intervention, an active coping strategy	One session per week	Four weeks	Not reported	No intervention	Job satisfaction<br><br>It was assessed using the 5-item General Satisfaction subscale of the Job Diagnostic Survey	Job satisfaction did not differ between intervention and control groups
Kuske 2009 [101]	Germany	Caregivers of nursing homes	Cluster RCT	Post-intervention and six months after completion of intervention	Mean age 44 years	94% were women	N = 38 training program in dementia care and N = 30 relaxation traing	28	96	One group received a training program in dementia care to improve caregivers' knowledge and competencies.<br><br>The second group received a relaxing training	An hour weekly session of training program in dementia care.<br><br>A 1-h weekly session of relaxation training such as muscle relaxation, breathing relaxation or guided imaginary journeys	13 weeks	During work hours	No intervention	Burnout<br><br>Burnout was measured with the Maslach Burnout Inventory	Emotional exhaustion, depersonalization and personal accomplishment did not differ between the groups
Tveito 2009 [56]	Norway	Nurses working in a nursing home for older people	Individually RCT	Post-intervention and one year follow-up (for sickness absence only)	Mean age 42.8 years	Women only	19	21	40	An integrated health program consisting of exercise, stress management, health information and an examination of workplace to cope with the job	(1) one hour of aerobic dancing three times a week, (2) an hour weekly session (total 15 h) of education about stress, coping, health and lifestyle, and (3) an examination of the workplace on how to	9 months	During work hours	No intervention	Sickness absence, general health, demands, control, and effort reward imbalance<br><br>Register data on sickness absence	Sickness absence, general health, demands, control, and effort reward imbalance did not differ between intervention and control groups at post-intervention

											organize and cope with the job				for one year after intervention<br><br>General health was assessed using the 5-item of the generic health status inventory SF-36<br><br>Psychological demands were measured using the 5-item from short version of the Psychological Demands dimension from the demand/control model<br><br>Control was measured using the 6-item from short version of the Decision Latitude dimension from the demand/control model	The intervention also had no effect on sickness absence at 1-year follow-up
Hugenholtz 2008 [106]	The Netherlands	Occupational physicians	Cluster RCT	Post-intervention and 7 months after baseline (3 months after the completion of the intervention)	Mean age 48 ± 5.8 for intervention group and 45 ± 6.7 for control group	41.7% were women	49	59	108	An evidence-based medicine training program consisting of a course and case method learning sessions	The course consisted of three half-days within two weeks.<br><br>A 1- to 1.5- hour case method learning session every two weeks (total 10 sessions)	4 months	Not reported	No intervention	Job satisfaction and professional performance<br><br>Job satisfaction was assessed using the 7 of the 13 subscales of the physician work-life survey<br><br>Professional performance was assessed using self-reported information on	The intervention had effect on professional performance ($p < 0.001$)<br><br>Job satisfaction did not differ between intervention and control groups

															practice of keeping up-to-date and using knowledge in daily practice	
Peterson 2008 [54]	Sweden	Healthcare workers, social workers, service staff, administrators, teachers, and technicians with burnout (exhaustion burnout score above the 75th percentile)	Individually RCT	Post-intervention, 7 and 12 months after intervention	Mean age 52.7 ± 5.6 for intervention group and 50.7 ± 6.7 for control group	96.1% of intervention group and 83.8% of control group were women	51	80	131	A working peer-support group for stress and burnout	A 2-h weekly session and a follow-up meeting 4 weeks after completion of first 10 sessions	10 weeks	During work hours	No intervention	Burnout, perceived general health, quantitative demands, and work participation<br><br>Burnout was measured using the 16-item Oldenburg Burnout Inventory and consists of two subscales (exhaustion and disengagement)<br>General health was assessed by the five items of the Short Form health survey<br><br>Quantitative demands such as overtime and workload were assessed using the General Nordic Questionnaire for Psychological and Social Factors at Work<br><br>Work participation was assessed using a single question	Perceived general health ($p = 0.010$) improved and quantitative demands ($p = 0.014$) decreased at 12-month follow-up in the intervention group compared with control group<br><br>Exhaustion and disengagement did not differ between two groups at 12-month follow-up<br><br>Work participation was higher in intervention group compared with control group at 12-month follow (between-group difference 16.2%, 95% CI 5.2–29.7%)
Warming 2008 [138]	Denmark	Nurses	Cluster RCT	12 months	Mean age 34.8 years	90% were women	N = 55 for transfer technique and N = 50 for transfer technique plus physical	76	181	Education of patient transfer technique.<br><br>Education of patient transfer technique plus	Two nurses from each ward received a 4-day training for transfer technique and then they introduced transfer technique to their colleagues for two 6-week periods.	8 to 12 weeks	Education of transfer technique during work hours and 50% of physical fitness	No intervention	Sickness absence<br><br>Self-reported information on sickness absence due to low back pain during 12-month follow-up	Sickness absence did not differ between intervention and control groups

							fitness training			physical fitness training	1-h supervised aerobic fitness and strength training twice a week for 8 weeks		training during work hours			
Yamagishi 2008 [129]	Japan	Shift-working nurses	Individually RCT	3 weeks	Mean age 33.0 ± 7.1 years	Not reported	26	25	51	A web-based career identity training for stress management	A 2-h training session	One session only	At hospital or home	No intervention	Workload, job control and reward from work They were assessed using the 20-item Job Stress Brief Questionnaire	Workload, job control and reward from work did not differ between intervention and control group
Le Blanc 2007 [77]	The Netherlands	Oncology healthcare workers	Cluster RCT	6 and 12 months after the baseline	Mean age 36.2 ± 8.4 years	Probably 72% were women	260	404	664	A team-based support group meetings to discuss and identify solutions for job stressors	A 3-h monthly session	6 months	Workplace	No intervention	Burnout<br><br>Emotional exhaustion and depersonalization were assessed using the Dutch version the Maslach Burnout Inventory—Human Services Survey	Emotional exhaustion was lower in intervention group than control group at both 6- and 12-month follow-ups<br><br>Depersonalization was lower in intervention group than control group at 6-month follow-up but not at 12-month
Roelofs 2007 [133]	The Netherlands	Home care workers with current non-specific low back pain or history of 2 or more episodes of low back pain in the past 12 months	Individually RCT	12 months	19–62 years. Mean age 41.8 ± 9.7 for intervention group and 41.5 ± 9.8 for control group	97.8% were women	183	177	360	A lumbar support plus usual care	Wearing a lumbar support on working days	12 months	Workplace	Control group received a short course on healthy working methods and usual care	Sickness absence<br><br>Register data on the number of sickness absence days for 12 months follow-up<br><br>Self-reported data on the number of sickness absence days due to low back pain for 12 months follow-up	The number of registered all-cause sickness absence days did not differ between intervention and control groups (difference −5.0 days, 95% CI −21.1 to 6.8 days, *p* = 0.45)<br><br>The number of self-reported sickness absence days due to low back pain was lower in

																intervention group than control group (difference −4.8 days, 95% CI −6.2 to −2.2 days, p = 0.003)
Mackenzie 2006 [63]	Canada	Nurses and nurse aides	Individually RCT	4 weeks	Mean age 48.6 ± 6.5 for intervention group and 44.8 ± 8.2 for control group	96.7% were women	16	14	30	A brief mindfulness-based stress reduction intervention	A 30-min weekly group session. The intervention group was instructed to practice for at least 10 min per day, 5 days per week	4 weeks	Workplace	No intervention	Burnout and Job satisfaction<br><br>Burnout was measured by the Maslach Burnout Inventory<br><br>Job satisfaction was assessed using the Job Satisfaction Scale	The intervention reduced emotional exhaustion, and depersonalization<br><br>Personal accomplishment, and job satisfaction did not differ between intervention and control groups
Oman 2006 [94]	USA	Health professionals	Individually RCT	8 and 19 weeks	26–70 years. 72% aged 40–59 years	86% were women	27	31	58	A spiritually based passage meditation	2 h per week	8 weeks	Not reported	No intervention	Burnout, and Job satisfaction<br><br>Burnout was measured by the Maslach Burnout Inventory<br><br>Job satisfaction was measured using a single item	Emotional exhaustion, depersonalization, personal accomplishment, and job satisfaction did not differ between intervention and control groups at 8- or 19-week follow-up
Pryce 2006 [57]	Denmark	Healthcare workers of a psychiatric hospital	Cluster RCT	20 months	Mean age 43 years	92% were women	86	91	177	An open-rota intervention to improve work scheduling.<br><br>Intervention consisted of workshop on work scheduling and then employees developed, implemented, and managed an open-rota	A 1-day workshop	20 months	Workplace	No intervention	General health and Job satisfaction<br><br>Self-rated general health was measured using a single item<br><br>Job satisfaction was assessed using a five-item scale	The intervention improved job satisfaction (p < 0.01) but had no effect on self-rated general health

										scheduling system for their team						
Brox 2005 [134]	Norway	Employees of a community nursing home	Individually RCT	6 months	Mean age 42.5 years	97% of intervention group and 96% of control group were women	63	56	119	Intervention consisting of light aerobic, strengthening and stretching exercises	An hour session per week.<br><br>Classes of physical exercise, nutrition, and stress management were also offered	6 months	Workplace	No intervention	Sickness absence<br><br>Register data on sickness absence	Number of self-certified and doctor-certified sickness absences did not differ between intervention and control groups during the follow-up period
Cohen-Katz 2005 [69]	USA	Nurses	Individually RCT	Post-intervention	32–60 years. Mean age 46 years	Women only	14	13	27	A mindfulness-based stress reduction intervention	A 2.5-h weekly session, a full day (6 h) retreat and home practices for 6 days per week	8 weeks	Not reported	No intervention	Burnout<br><br>Burnout was measured by the 22-item Maslach Burnout Inventory	Emotional exhaustion reduced (p = 0.050) and personal accomplishment improved (p = 0.014) more in intervention group than control group<br><br>Depersonalization reduced more in intervention group than control group, but the difference did not reach statistical significance (p = 0.063)
Lökk 2000 [103]	Sweden	Nurses and nursing aides of two geriatric wards	Cluster RCT	Post-intervention and 10 weeks after the intervention (30 weeks from baseline)	20–62 years. Mean age 39 years for intervention and 37 years for control group	96.2% were women	14	12	26	Management change on psychosocial factors consisting of an initial education about stressors and stress management followed by practical problem-solving discussions	1-h once a week. Due to work schedules, every participant was able to attend the meetings once every two weeks (total 10 sessions)	20 weeks	Workplace	A routine staff meeting once a week	Burnout, job demands and job control.<br><br>Job demands and job control were measured using a validated questionnaire<br><br>Burnout was assessed using a single question	Work demand increased in intervention ward compared to control ward at 30-week follow-up but not at 20-week follow-up<br><br>Work control and burnout did not differ between intervention and

																control wards at 20- and 30-week follow-up
Gundewall 1993 [131]	Sweden	Nurses and nursing aides of a geriatric hospital	Individually RCT	13 months	18–58 years	Women (there was only one man)	28	32	60	Strengthening, endurance, and coordination exercises	On average 6 times per month	During follow-up period	During work hours	No intervention	Number of persons on sick leave due to a back complaint	1 of 28 participants in intervention group and 12 of 32 controls had been on sick leave.<br><br>Estimated RR 0.10 (CI 0.01–0.69)
Linton 1989 [135]	Sweden	Nurses and nursing aides with back pain	Individually RCT	6 months	20–59 years. Median age 43 years	Women only	36	30	66	Physical and behavioral preventive intervention	8 h a day on weekdays including various exercises (walking, swimming, jogging, cycling, etc.) for 4 h/day, ergonomic education, and behavioral therapy twice a week	5 weeks	During work hours	No intervention	Sickness absence<br><br>Register data on sickness absence for 6 months follow-up	Number of sickness absence days due to musculoskeletal problems or other illnesses did not differ between intervention and control groups
